# Polycystic subdural hygroma associated with immunoglobulin G4-related intracranial hypertrophic pachymeningitis: a case report

**DOI:** 10.1186/s12883-020-01815-z

**Published:** 2020-06-04

**Authors:** Kazumichi Ota, Yoshihiko Nakazato, Risa Okuda, Ryu Yokoyama, Hitoshi Kawasaki, Naotoshi Tamura, Toshimasa Yamamoto

**Affiliations:** Department of Neurology, Saitama Medical University, 38 Morohongo Moroyama, Iruma-gun, Saitama, 350-0495 Japan

**Keywords:** Hypertrophic pachymeningitis, IgG4-related disease, Polycystic hygroma, Hematoma

## Abstract

**Background:**

Recent studies have examined hypertrophic pachymeningitis as an IgG4-RD. However, there are no reports of immunoglobulin G4 (IgG4)-related hypertrophic pachymeningitis with polycystic subdural hygroma.

**Case presentation:**

A 56-year-old man presented to the hospital with complaints of a persistent, pulsatile, occipital headache and general malaise. Magnetic resonance imaging of the brain revealed hypertrophic pachymeningitis with polycystic subdural hygroma and hematoma. Based on the dural biopsy findings and exclusion of other diseases, the patient was diagnosed with immunoglobulin G4 (IgG4)-related hypertrophic pachymeningitis. IgG4-related diseases may cause subdural hygroma more commonly than other diseases that cause hypertrophic pachymeningitis.

**Conclusions:**

This is the first case report discussing polycystic subdural hygroma and hematoma with IgG4-related hypertrophic pachymeningitis.

## Background

Immunoglobulin G4-related disease (IgG4-RD) was first reported as hyper-IgG4emia in autoimmune pancreatitis [1]. In recent studies, the hypertrophic pachymeningitis spectrum has also been included in IgG4-RDs [2–4]. Several studies have been conducted to examine the clinical and imaging features of IgG4-related hypertrophic pachymeningitis [2–4]. However, there are no reports of IgG4-related hypertrophic pachymeningitis with polycystic subdural hygroma. This is the first case report of a polycystic subdural hygroma and hematoma with IgG4-related hypertrophic pachymeningitis.

## Case report

**Fig. 1:**
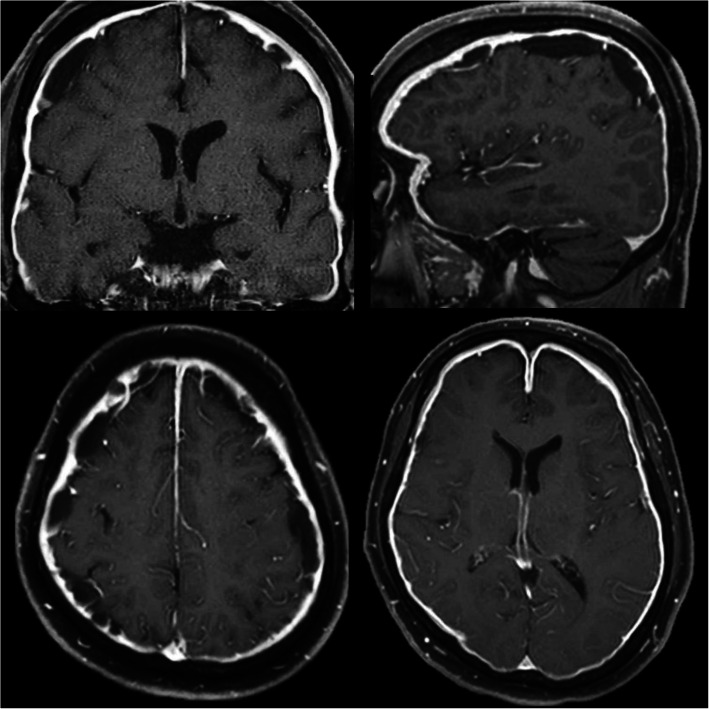
Brain MRI (gadolinium enhanced T1-wighted images) on admission. MRI revealed polycystic subdural hygroma, hematoma, and diffuse thickening of the dura mater when probed using contrast enhancement (MRI, magnetic resonance imaging)

**Fig. 2:**
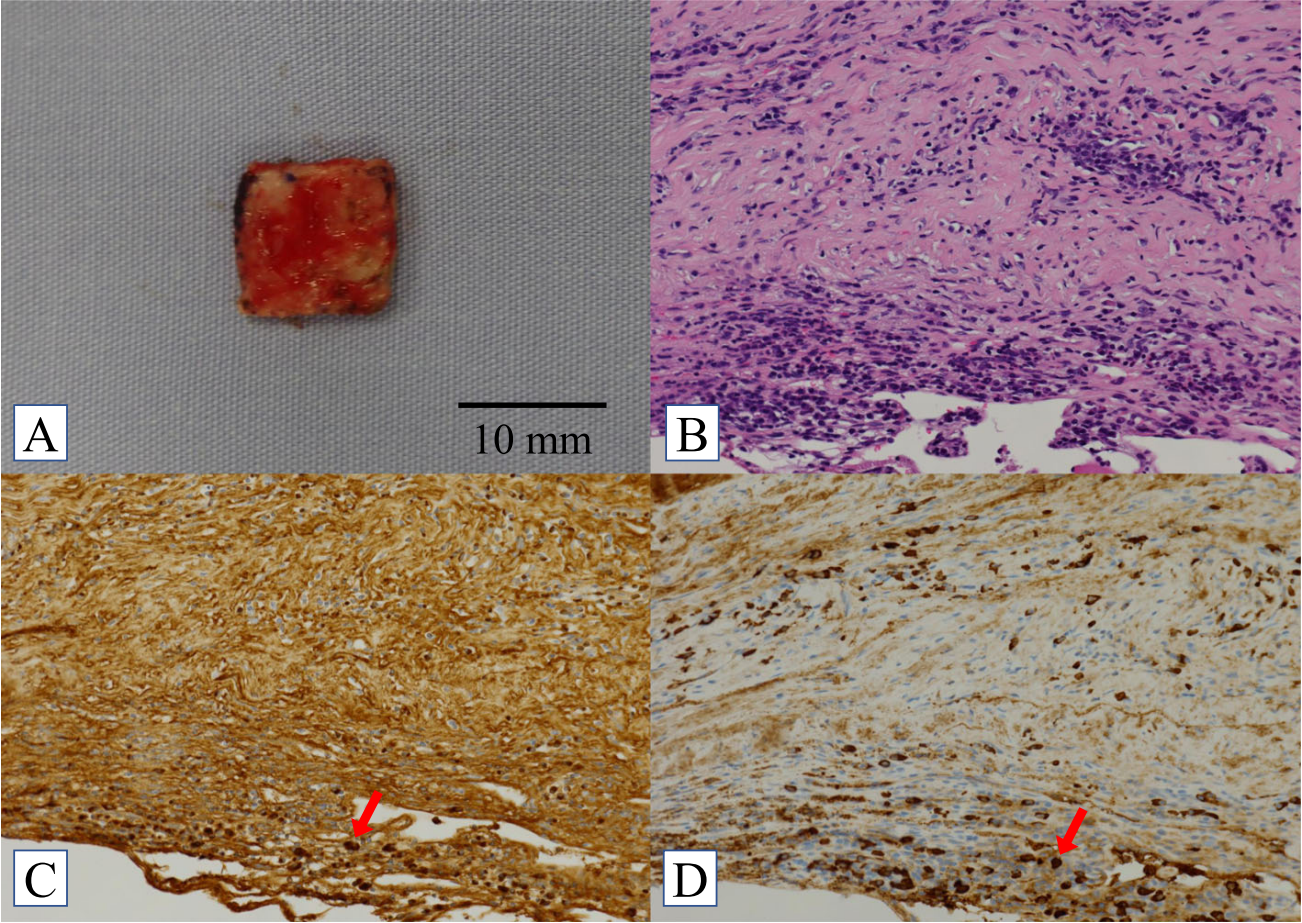
Pathological findings. a: Macroscopic observation shows that the dura surface was red and edematous, and very fragile. b: The photomicrograph shows that the solid lesion consisted of proliferated fibroblast cells and infiltrated mononuclear cells, including predominantly plasma cells, with abundant collagenous tissue (H&E; original magnification, × 400). c and d: IgG(C) and IgG4(D) staining revealed that most of the plasma cell infiltration into sclerosing lesions were IgG positive (IgG4-positive plasma cell/high power field, 60), and the ratio of IgG4-positive plasma cells (arrow) to the overall IgG-positive cells (arrow) is greater than 50% in the area containing the highest infiltration of plasma cells (original magnification, × 400) (H&E, hematoxylin and eosin; IgG, immunoglobulin G)

A 56-year-old man with a history of asthma, sinusitis, serous otitis media, idiopathic eosinophilia, recurrent idiopathic myocarditis, and idiopathic interstitial pneumonia was treated with prednisolone (PSL) at a dose of 27.5 mg/day. He had no symptom with steroid therapy. He complained of a persistent, pulsatile, occipital headache, and general malaise 2 weeks before admission, when the dose of PSL was reduced from 27.5 mg/day to 22.5 mg/day. However, he did not exhibit symptoms such as fever, weight loss, joint pain, rash, or diarrhea. A neurological examination revealed paralysis of the left abducens nerve. Magnetic resonance imaging (MRI) of the brain showed polycystic subdural hygroma and hematoma. In addition, with contrast enhancement, a diffuse thickening of the dura mater was observed (Fig. 1). Whole-body computed tomography (CT) scans showed no signs of lymphadenopathy or malignant tumors. Gallium scintigraphy revealed diffuse accumulation in the kidneys; however, there was no accumulation in the lymph nodes and joints. Urinalysis showed a tubular defect (N-acetyl-beta glucosamines 45 IU/L, beta-2-microglobulin 9156 μg/L), suggesting interstitial nephritis. Microbial analyses and immunologic bioassays were uninformative. There was no complement consumption or eosinophilia. His serum IgG4 level was slightly increased (126 mg/dL) at the PSL dose of 22.5 mg/dL. However, one year prior to the study, it was higher (332 mg/dL), although the dose of PSL was 7 mg/day. Craniotomy was performed from the left side of the head with a burr hole, made a dural incision, and collected a sample. After biopsy and dural suture, it was confirmed that was no leakage of spinal fluid by the Valsalva test, preventing low intracranial pressure syndrome from occurring. Dural biopsy confirmed that the dura was 2 mm thick, and its surface was red and edematous, and very fragile. Histopathological examination of the surgical specimen revealed a fibro-inflammatory lesion in the thickened dura due to lymphoplasmacytic infiltrates. Immunohistochemical analysis showed lymphocytic infiltration, 60 IgG4+ plasma cells per high-power field, and an IgG4+/IgG ratio greater than 50% (Fig. 2). There was no evidence of a malignant tumor, histiocytic disease, or granulomatous disease. Staining for microbial pathogens was negative. We established a diagnostic of IgG4-RD based on clinical diagnostic criteria [5, 6], we established a diagnosis of IgG4-RD; the unexplained multiple organ disorders (as mentioned before) also suggested IgG4-RD. The symptoms improved quickly with oral PSL therapy (45 mg/day, 0.6 mg/kg/day) after 2 courses of methylprednisolone 1 g pulse therapy for 3 days, and the subsequent brain MRI showed complete regression of meningeal thickening, polycystic subdural hygroma, and hematoma. Thereafter, tacrolimus (3 mg/day) was added, and the PSL dose was reduced by 2.5–5 mg every two weeks to a final dose of 10 mg/day. The patient had no recurrence of headache with oral treatment using PSL (10 mg/day) and tacrolimus (3 mg/day), and his serum IgG4 level decreased to 9.6 mg.

## Discussion and conclusions

Intracranial hypertrophic pachymeningitis rarely accompanies subdural hygroma or hematoma. Only three cases have been reported in the literature [7, 8], including our case, of which two cases involved a diagnosis of IgG4-RDs. Until now, there have been no reports of IgG4-RDs associated with polycystic subdural hygroma or hematoma. In our case, exudative retention due to strong inflammation of the dura may have caused reactive film formation bleeding from the capsule or bridging vein [7]. The dura adhesion may indicate that the exudate enters the space between the membranes and forms a cyst. In conclusion, we suggest that IgG4-RDs may often cause subdural hygroma.

## Data Availability

The datasets used and/or analyzed during the current study available from the corresponding author on reasonable request.

## Abbreviations

IgG4: Immunoglobulin G4
IgG4-RD: Immunoglobulin G4-related disease
PSL: Prednisolone
MRI: Magnetic resonance imaging
CT: Computed tomography
H&E: Hematoxylin and eosin
